# Further support linking the 22q11.2 microduplication to an increased risk of bladder exstrophy and highlighting *LZTR1* as a candidate gene

**DOI:** 10.1002/mgg3.666

**Published:** 2019-05-01

**Authors:** Johanna Lundin, Ellen Markljung, Izabella Baranowska Körberg, Wolfgang Hofmeister, Jia Cao, Daniel Nilsson, Gundela Holmdahl, Gillian Barker, Magnus Anderberg, Vladana Vukojević, Anna Lindstrand, Agneta Nordenskjöld

**Affiliations:** ^1^ Department of Women’s and Children’s Health Karolinska Institutet Stockholm Sweden; ^2^ Department of Clinical Genetics Karolinska University Hospital Stockholm Sweden; ^3^ Department of Molecular Medicine and Surgery Karolinska Institutet Stockholm Sweden; ^4^ Science for Life Laboratory Karolinska Institutet Science Park Stockholm Sweden; ^5^ Department of Pediatric Surgery Sahlgrenska Academy Gothenburg Sweden; ^6^ Department of Women’s and Children’s Health Uppsala University Uppsala Sweden; ^7^ Department of Pediatric Surgery University Hospital Lund Lund Sweden; ^8^ Department of Clinical Neuroscience, Center for Molecular Medicine Karolinska Institutet Stockholm Sweden; ^9^ Pediatric Surgery Astrid Lindgren Children Hospital, Karolinska University Hospital Stockholm Sweden

**Keywords:** array‐CGH, bladder exstrophy, confocal microscopy, exome sequencing, fluorescence spectrometry, LZTR1, microduplication

## Abstract

**Background:**

The bladder exstrophy‐epispadias complex (BEEC) is a congenital malformation of the bladder and urethra. The underlying causes of this malformation are still largely unknown; however, aside from environment, genetics is thought to play an essential role. The recurrent 22q11.2 microduplication is the most persistently detected genetic aberration found in BEEC cases.

**Methods:**

We performed array comparative genomic hybridization (array‐CGH) analysis of 76 Swedish BEEC patients. Statistical analysis was performed on current dataset pooled with previously published data on the 22q11.2 microduplication in BEEC patients. We performed massive parallel sequencing (MPS) of the 22q11.2 region in 20 BEEC patients without the 22q11.2 microduplication followed by functional studies.

**Results:**

We identified three additional cases with the 22q11.2 microduplication. Pooling data from this study with previously published reports showed a statistically significant enrichment of the 22q11.2 microduplication in BEEC patients (2.61% in cases vs. 0.08% in controls; OR = 32.6; *p* = 8.7 × 10^−4^). MPS of the 22q11.2 region in 20 BEEC patients without the 22q11.2 microduplication identified a novel variant in *LZTR1* (p.Ser698Phe) in one patient. Functional evaluation of the *LZTR1* p.Ser698Phe variant in live NIH 3T3 cells showed that the concentration and cytoplasmic mobility differ between the Lztr1_wt_ and Lztr1_mut_, indicating a potential functional effect of the LZTR1_mut_.

**Conclusion:**

Our study further emphasizes the involvement of the 22q11.2 region in BEEC development and highlights *LZTR1* as a candidate gene underlying the urogenital malformation.

## 1 INTRODUCTION

Bladder exstrophy is a congenital urogenital malformation and part of a clinical spectrum of the bladder exstrophy‐epispadias complex (BEEC, OMIM 600057). The phenotypic severity varies from isolated epispadias, through to classic bladder exstrophy and the most severe form, cloaca exstrophy. The reported European incidence of BEEC is 1 in 30,000 live births while classic bladder exstrophy occurs in about 1 in 46,000 live births. The incidence of classic bladder exstrophy in Sweden is reported to be about 1 in 28,900 live births (Cervellione et al., 2015). Most cases are sporadic; however, an increased risk in siblings has been described. About 3% of all cases are classified as familial with at least two affected cases in the same family (Reutter, Shapiro, & Gruen, 2003). The recurrence risk for a sibling is 1 per 70 live births, which is equivalent with a 500 times increased risk in siblings compared to the general population (Shapiro, Lepor, & Jeffs, 1984). Furthermore, pairwise concordance rates among monozygotic (MZ) and dizygotic (DZ) BEEC twin pairs are 45% for MZ and 6% for DZ twins respectively (Reutter et al., 2007). These combined data suggest an underlying genetic component.

From studies both on chromosomal as well as base‐pair level several regions, genes and gene‐pathways have been suggested to be associated with BEEC susceptibility (Arkani et al., 2018; Baranowska Korberg et al., 2015; Draaken et al., 2015; Reutter et al., 2014; von Lowtzow et al., 2016). For example, a 900 kb microduplication on chromosome 19p13.12 was identified in one patient (Draaken et al., 2013). The duplication was confirmed de novo and one gene in the duplicated region, the *Wiz* gene, showed specific expression in cloaca and rectum regions in mice. Several studies have suggested the *WNT* family of genes to be involved in BEEC (Draaken et al., 2015; Qi et al., 2011; Reutter et al., 2014)**. **We previously evaluated the *WNT*‐pathway genes in 20 BEEC cases by massive parallel sequencing (MPS). In total 13 variants were identified in *WNT*‐pathway genes of which seven variants were novel. One de novo variant in the *WNT3 *(OMIM 165330) gene was further evaluated in zebrafish supporting an altered function of the mutant (Baranowska Korberg et al., 2015). Also, *Wnt3* and *Wnt9b* (OMIM 602864) have been shown to be expressed in genital tubercle in mouse embryos in a period that corresponds to bladder development in humans.

Most chromosomal regions, genes and pathways have been identified in isolated cases or by association studies. The only recurrent and most frequently detected genetic aberration found in BEEC cases is the recurrent 3 Mb large tandem 22q11.2 microduplication (Draaken et al., 2014,2010; Lundin et al., 2010; Pierquin & Uwineza, 2012). We previously screened 36 patients born with bladder exstrophy for copy number variants and reported two unrelated cases carrying the 22q11.2 microduplication (one de novo and one inherited) (Lundin et al., 2010). Both cases were also diagnosed with hearing impairment, leading us to speculate of a possible connection of these two phenotypes. Our finding has been independently confirmed by three other studies where a 22q11.2 microduplication has been identified in an additional seven bladder exstrophy cases out of 275 analyzed in total (Draaken et al., 2014,2010; Pierquin & Uwineza, 2012).

Chromosomal rearrangements affecting the 22q11.2 region are implicated in several human genetic disorders like the DiGeorge syndrome (DGS)/velocardiofacial syndrome (VCFS) (OMIM 188400 and OMIM 192430, respectively) and cat‐eye syndrome (OMIM 115470). The 22q11.2 microdeletion syndrome, DGS/VCSF, is the most common with an estimated incidence of 1 in 4,000 to 1 in 6,000 live births (Botto et al., 2003; Devriendt, Fryns, Mortier, van Thienen, & Keymolen, 1998). Duplications of 22q11.2.2 are less frequently observed but are in fact more common (Van Campenhout et al., 2012; Portnoi, 2009). The reason the duplication is less frequently detected is that carriers of a 22q11.2 microduplication present with a variable phenotype ranging from healthy to severe developmental disabilities and congenital malformations. Furthermore, the duplication is often inherited from a healthy parent and variability in the clinical severity and overall expression is seen also within the same family (Alberti et al., 2007; Courtens, Schramme, & Laridon, 2008; Edelmann et al., 1999; Ensenauer et al., 2003; Hassed, Hopcus‐Niccum, Zhang, Li, & Mulvihill, 2004; Portnoi, 2009; Portnoi et al., 2005; Wentzel, Fernstrom, Ohrner, Anneren, & Thuresson, 2008; Yobb et al., 2005).

The 22q11.2 region contains several genes that most likely contribute in different ways to the diverse phenotype associated with the 22q11.2 microdeletion/microduplication syndromes. Draaken et al. identified a BEEC phenocritical 22q11.2 region containing 12 genes. These were evaluated using whole‐mount in situ hybridization (WISH) in mouse embryos at gestational week 9.5. This time‐point corresponds to human gestational week 4 representing the critical timeframe for the initial stages of external genital formation. Four genes; *PI4KA *(OMIM 600286),* SNAP29 *(OMIM 604202),* CRKL *(OMIM 602007) and* LZTR1* (OMIM 600574) showed ubiquitous expression. From this data together with that from Molecular Anatomy Project (http://www.gudmap.org/), reporting expression of *CRKL* in mouse kidney at E14.5 and expression of *THAP7* (OMIM 609518) in the genitourinary tract at E10.5, the authors suggested *CRKL*, *THAP7* and *LZTR1* as possible candidate genes for the BEEC phenotype (Draaken et al., 2014).

The *LZTR1* gene belongs to the BTB‐kelch superfamily and is located within the recurrent 22q11.2 microdeletion/microduplication region (Nacak, Leptien, Fellner, Augustin, & Kroll, 2006). The BTB‐kelch superfamily plays important roles during fundamental cellular processes, such as the regulation of cell morphology, migration, and gene expression. Most BTB‐kelch proteins co‐localize with actin and have a role in cytoskeleton stabilization and organization. However, Nacak et al. showed that *LZTR1* encodes a Golgi matrix‐associated protein, which indicates that it is not involved in the organization and stabilization of the cytoskeleton and most likely not acting as a transcriptional regulator (Nacak et al., 2006). The exact function of the *LZTR1* protein is still largely unknown.

The aim of this study was to search for the 22q11.2 microduplication in an unpublished cohort of Swedish BEEC patients and to further investigate the 22q11.2 region in cases without the microduplication as well as to functionally evaluate identified candidate variants.

## 2 MATERIAL AND METHODS

### 2.1 Ethical compliance

The study was approved by the local ethics committee and conforms to the Declaration of Helsinki standards. All patients gave their informed consent prior to inclusion in the study.

### 2.2 Patients and controls

All BEEC patients were recruited from the Pediatric Surgery Departments in Stockholm, Gothenburg, Uppsala and Lund, Sweden. DNA samples from 362 placentas, acquired after normal delivery of healthy new‐borns, and 740 anonymous blood donors were used as controls. All control samples were collected at the Karolinska University Hospital, Stockholm, Sweden.

### 2.3 Array‐CGH analysis

A 180K custom oligonucleotide microarray with whole genome coverage and a median probe spacing of approximately 18 kb was used (OxfordGeneTechnology, Yarnton, Oxfordshire, UK). This array design is used as a routine diagnostic tool at the Department of Clinical Genetics, Karolinska University Hospital. Stockholm, Sweden. Genomic DNA isolated from blood and sex‐matched reference DNA isolated from healthy controls (Promega, Madison, WI) was analyzed. Sample labeling (CGH labeling kit for oligo arrays, Enzo Life Sciences, Farmingdale, NY), hybridization and slide washing (Oligo aCGH/ChIP‐on‐Chip Wash Buffer Kit, Agilent Technologies, Wilmington, DE) were performed according to the manufacturers’ recommendations. Slides were scanned using the Agilent Microarray Scanner (G2505C, Agilent technologies, USA) with 3 mm resolution. Raw data were normalized using Feature Extraction Software (Agilent Technologies, USA), and log2 ratios were calculated by dividing the normalized intensity in the sample by the mean intensity across the reference sample. The log2 ratios were plotted and segmented by circular binary segmentation in the CytoSure Interpret software (Oxford Gene Technology, Oxfordshire, UK). Oligonucleotide probe positions were annotated to the human genome assembly hg19 (www.genome.ucsc.edu).

### 2.4 Massive parallel sequencing

Massive parallel sequencing (MPS) was performed on 20 BEEC patients without the 22q11.2 duplication. BEEC cases with already collected parental samples were chosen to enable confirmation and family segregation analyses. Libraries for sequencing on Illumina HiSeq2000 (Illumina) were prepared from DNA samples and exome sequences were enriched with Agilent SureSelect Human All Exon 50M (Agilent), according to manufacturer's instructions. Postcapture libraries were sequenced as 2 × 100 bp paired end reads on the Illumina sequencer. Reads were base called using Illumina OLB (v 1.9, Illumina). Sample library preparation, sequencing, and initial bioinformatics up to base calling and demultiplexing were performed at the Science for Life Laboratory, Stockholm.

An in‐house pipeline was used to process reads, call variants and annotate them. It is freely available under a GPL license (http://github.com/dnil/etiologica). Briefly, reads were mapped to the human reference genome (hg19) using Mosaik (v1.0.1388) (Michael Strömberg, unpublished, http://code.google.com/p/mosaik-aligner/). Duplicate read pairs were removed using Mosaik DupSnoop. Variants were called using the samtools package (v.0.1.18) (Li et al., 2009), quality filtered (Q ≥20) and further annotated using ANNOVAR (version 2012 May 25) (Wang, Li, & Hakonarson, 2010) to estimate allele frequencies in large databases, predict, pathogenicity, evolutionary conservation, and segmental duplication status.

### 2.5 Sanger sequencing and TaqMan SNP genotyping

Sequencing was performed using the PCR primers and Big Dye® terminator v3.1 cycle sequencing kit (Applied Biosystems) according to standard protocol. Size separation was performed on ABI 3730 DNA Analyzer (Applied Biosystems) and electropherograms analyzed using the CodonCode Aligner software package (CodonCode Corporation, Denham, MA, USA). The protein coding region of *LZTR1* (NM_006767.3) all 21 exons and the 950 base pair region upstream of exon 1 were PCR amplified in 95 BEEC patients. All variants identified by MPS were confirmed using traditional Sanger sequencing. Primer sequences and PCR conditions are available upon request.

A custom TaqMan® SNP genotyping assay for the *LZTR1* mutation was designed using the online tool and ordered from Life Technologies Corporation. A total of 362 placenta control samples and 740 anonymous blood donors were analyzed using standard protocol. The ABI 7900HT instrument and the SDS software (v2.4, Applied Biosystems) were used for data collection and analysis.

### 2.6 Reporter constructs, cell culturing, and transfection

*LZTR1* (NM_006767.3) human cDNA ORF construct tagged with C‐termial GFP‐tag in pCMV6‐AC‐GFP vector was purchased (RG204432, Origene, MD, USA). Plasmid was cloned, transformed and grown according to manufacturer's instructions (One Shot^®^ MAX Efficiency^®^ DH10B‐T1^®^ Competent Cells, Invitrogen, Carlsbad, CA, USA). Site‐directed mutagenesis (GENEART Site‐Directed Mutagensis System, Invitrogen) of the wild type construct was performed to generate the mutant *LZTR1c.2093C>T*, followed by sequencing of the entire insert. Plasmids were purified with either Plasmid Mini Kit or Endo‐Free Maxi prep (Qiagen, Hilden, Germany). Sequences were analyzed using the CodonCode Aligner software package (CodonCode Corporation, MA, USA).

Mouse adherent fibroblasts, NIH 3T3 (ATCC® CRL‐1658^TM^) were purchased and grown according to manufactures recommendation in Dulbecco's modified eagle's medium (DMEM) supplemented with 10% bovine calf serum and 0,5% Penicillin‐Streptomycin (ATCC, Wesel, Germany). Cells were tested negative for mycoplasma (Venor GeM Mycoplasma Detection Kit, Minerva Biolabs, Berlin, Germany).

Transient transfection was performed at approximately 60%–70% confluency on 8‐well Nunc™ Lab‐Tek™ Chambered Coverglass (Thermo Scientific, MA, USA) in triplicate. Transfections were performed using Xfect^TM^ Transfection Reagent according to manufactures recommendations (Clontech, CA, USA).

### 2.7 Confocal Laser Scanning Microscopy (CLSM) and Fluorescence Correlation Spectroscopy (FCS)

CLSM imaging and FCS measurements were performed using the LSM510 ConfoCor3 instrument (Carl Zeiss, Jena, Germany) individually modified to enable fluorescence imaging with silicone avalanche photodiodes (SPCM‐AQR‐1X, PerkinElmer, USA) (Vukojevic et al., 2008). These single‐photon detectors are characterized by high photon detection efficiency ([50%–72%] % in the 500–850 nmol L^‐1^ range) and low noise levels (<200 Hz), which makes it possible to visualize fluorescent molecules at very low concentrations using low excitation intensities, which is favorable for live cell imaging. The C‐Apochromat 40×, NA = 1.2, water immersion UV‐VIS‐IR objective was used throughout. The Green Fluorescent Protein (TurboGFP) was excited using the 488 nmol L^‐1^ line of the Ar/ArKr laser. Emitted light was separated from the incident light using the main dichroic beam splitter HFT KP 700/488 and split using the secondary dichroic beam splitter NFT 545 in order to separate TurboGFP fluorescence from NIH 3T3 autofluorescence (Roederer & Murphy, 1986). Further spectral narrowing of the emitted light was achieved using emission filters in front of the detectors. For TurboGFP imaging, emitted light was collected using the band pass filter BP 505–530. Autofluorescence was visualized using the long pass filter LP 580. Images were acquired without averaging, using a scanning speed of 25.6 or 51.2 µs/pixel, and 512 × 512 or 1,024 × 1,024 pixels per frame. Pinhole of 1 Airy (70 µm) was used for both CLSM and FCS. The optical setting for FCS was identical as for CLSM, with the main dichroic beam splitter HFT KP 700/488 and the band pas filter BP 505–530 for TurboGFP, except that the light was not split in order to collect autofluorescence.

Fluorescence intensity fluctuations were recorded in arrays of 10 consecutive measurements, each measurement lasting 10 s and subjected to temporal autocorrelation analysis to extract information about the average number of Lztr1_wt_ and Lztr1_mut_ molecules in the observation volume element (N), that is, their concentration in live in NIH 3T3 cells, and their diffusion time (τ_D_). For more information on FCS and temporal autocorrelation analysis, we refer the interested readers to recent reviews (Elson, 2013; Vukojevic et al., 2005). Briefly, as a first step of temporal autocorrelation analysis the so‐called normalized autocorrelation function G(τ) was derived:

(1) $$G\left(\tau \right)=1+\frac{〈\delta I(t)\;\delta I(t+\tau )〉}{{〈I(t)〉}^{2}}$$

G(*τ*) gives the correlation between the deviation of fluorescence intensity measured at a certain time point *t*, *δ*I(*t*) = I(*t*)−‹I(*t*)›, which is given as the difference in fluorescence intensity I(*t*) and the mean fluorescence intensity over the whole time‐series (‹I(*t*)›), and the intensity in the lag version of the same time series, that is, the same time series shifted by a lag time (*τ*), *δ*I(*t* + *τ*) = I(*t* + *τ*)−‹I(*t*)›. G(*τ*) is then plotted as a function of lag time τ to yield the temporal autocorrelation curve (tACC). The tACCs were then fitted using theoretical model functions for free 3D diffusion with triplet contribution (Elson, 2013; Vukojevic et al., 2005).

(2) $$G\left(\tau \right)=1+\frac{1}{N}\cdot \left(\sum_{i}\frac{y_{i}}{\left(1+\frac{\tau }{\tau_{\mathrm{Di}}}\right)\sqrt{1+\frac{w_{\mathrm{xy}}^{2}}{w_{z}^{2}}\frac{\tau }{\tau_{\mathrm{Di}}}}}\right)\cdot \left[1+\frac{T}{1-T}\exp\left(-\frac{\tau }{\tau_{T}}\right)\right]$$

In Equation 2, *T* is the average equilibrium fraction of molecules in the triplet state; *τ*_T_ is the triplet relaxation time; *i* is the number of components, that is, chemical species that can be distinguished based on differences in diffusion; *τ*_D_ *_i_* is the diffusion time of the *i *‐th component and *y_i_* is its relative amplitude (∑*y_i_* = 1); *w_xy_* and *w_z_* are the radial and axial radii of a Gaussian beam profile at which the fluorescence intensity has dropped by a factor of e^2^ compared to its peak value. The parameter (*w_xy_*/*w_z_*)^2^ is determined by instrument calibration using a standard solution of a fluorescent molecule for which the diffusion coefficient is known. Here, aqueous solutions of rhodamine 6G (Rh6G) were used. The standard solution, 10–50 nmol L^‐1^, was freshly prepared every day. Rh6G diffusion time was determined to be τ_D_ = (27 ± 2) µs, and the so‐called structure parameter was determined to be *S^2^* = (*w_z_*/*w_xy_*)^2^ = (7 ± 1).

The dedicated ConfoCor3 Zeiss LSM software (Carl Zeiss, Jena, Germany) was used for temporal autocorrelation analysis and for fitting the experimentally derived tACCs. The simplest model, that is, the model with the lowest number of components (the lowest number of *i*) was always used. The tACC for TurboGFP could be fitted with a model for free 3D diffusion of one component, yielding a diffusion time of τ_D,TurboGFP_ = (220 ± 20) µs. Two characteristic times were observed for Lztr1_wt_, τ_D1_ = (340 ± 50) µs and τ_D2_ = (27 ± 3) ms. The relative contribution of the second fraction increased for increasing Lztr1_wt_ concentrations, ranging from *x*_2_ = (0.20 ± 0.05) at the lowest concentration (10 nmol L^‐1^) to *x*_2_ = (0.32 ± 0.05) at the highest concentration measured (70 nmol L^‐1^). For Lztr1_mut_, cytoplasmic signal and corresponding correlation curves were not observed. More than 25 cells were analyzed in each group.

## 3 RESULTS

### 3.1 Array‐CGH

Array comparative genomic hybridization (array‐CGH) analysis of 76 previously unpublished BEEC patients revealed three novel 22q11.2 microduplication carriers (Table 1). Patient 1 had a 2.57 Mb duplication at genomic position chr22:18,938,160–21,505,425 according to Hg19. This duplication was inherited from the healthy mother. Patient 1 had a medical history of hearing impairment and mild neuropsychiatric illness in addition to bladder exstrophy. Patient 2 had a 2.57 Mb duplication at genomic position chr22:18,890,264–21,464,056 according to Hg19. For patient 2, no parental samples were available for testing. Patient 2 had a medical history of, besides bladder exstrophy, neuropsychiatric illness. Patient 3 had a de novo 2.57 Mb duplication at genomic position chr22: chr22:18,890,264–21,461,788 according to Hg19. Patient 3 was a new‐born boy. The mother was medicated for Crohn's disease during pregnancy. The pregnancy was uneventful until week 30, when duodenal atresia and polyhydramniosis was detected on an ultrasound screen. The boy was born by spontaneous delivery in week 32 + 5 with normal birth weight. The duodenal atresia, linked to a pancreas annullare, was neonatally surgically corrected. Neonatal genetic investigation for aneuploidy of chromosomes 13, 18, 21, X and Y showed normal result. During follow‐up visit some dysmorphic features were noticed; single transverse palmar crease, high forehead, large eyes, protruding tongue, and glanular epispadias with a dorsal curvation.

**Table 1:** 22q11.2 microduplication cases

Case	Duplicated region	Inheritance	Phenotype
Patient 1	chr22:18,938,160–21,505,425	Maternal	Bladder exstrophy, hearing impairment, mild neuropsychiatric illness
Patient 2	chr22:18,890,264–21,464,056	n.a.^a^	Bladder exstrophy, neuropsychiatric illness
Patient 3	chr22:18,890,264–21,461,788	de novo	Glanular epispadia with dorsal curvation, duodenal atresia, single transverse palmar crease, high forehead, large eyes, protruding tongue

a Parental data was not available (n.a.).

### 3.2 Statistical analysis

To calculate the risk for BEEC development associated with the 22q11.2 duplication, we combined data on 22q11.2 duplication frequency in published BEEC cohorts (Draaken et al., 2014,2010; Lundin et al., 2010). Including the current, altogether four studies provided data on a total of 422 BEEC patients and 1,219 controls. There was a statistically significant enrichment of the 22q11.2 microduplication in BEEC patients (2.61% in cases compared to 0.08% in controls; OR = 32.6; 95% CI = 4.2–253.3; *p* = 8.7 × 10^−4^). In a published cohort of children with intellectual disability and various congenital defects (15,767 cases and 8,329 controls) the incidence of the 22q11.2 duplication was 0.32% with an estimated penetrance of 23% (Cooper et al., 2011). In conclusion, the 22q11.2 duplication is more common among patients with intellectual disability and various congenital defects compared to normal controls (0.32%; *p* = 1.3 × 10^−5^) and even more common among BEEC patients (2.61%: *p* < 0.00001).

### 3.3 Mutation screening

To evaluate the protein coding regions of genes in the 22q11.2 region, 20 BEEC patients without the 22q11.2 duplication were selected for MPS. A heterozygous missense variant in the *LZTR1* gene (NM_006767.3, c.2093C>T, p.Ser698Phe, rs760064852) was identified in one individual with isolated bladder exstrophy. The variant was confirmed by Sanger sequencing. Three different in silico prediction software packages; MutationTaster (Schwarz, Rodelsperger, Schuelke, & Seelow, 2010), SIFT (Kumar, Henikoff, & Ng, 2009) and PolyPhen‐2 (Adzhubei et al., 2010), were used to assess pathogenicity and all three software packages predicted the variant to be disease causing or probably damaging. The variant is in the BTB domain and a highly conserved amino acid. Family segregation showed that the variant was inherited from the patient's healthy mother. This variant has been seen in two cohorts in the UK10K project and submitted to dbSNP but no frequency data is available. The variant was not detected among the control samples. The variant was not reported in the ExAC dataset of 60,706 unrelated individuals (http://exac.broadinstitute.org/) or in the gnomAD dataset of 123,136 exomes and 15,496 genomes from unrelated individuals sequenced as part of various disease‐specific and population genetic studies (http://gnomad.broadinstitute.org/) (Lek et al., 2016).

To further explore the frequency of *LZTR1* variants in BEEC we Sanger sequenced the promoter region (950 bp upsteam of exon 1) and protein coding region (21 exons) of *LZTR1* gene in an additional 94 BEEC patients. The success rate was >95% of each exon. One variant of unknown significance was identified in one individual who was born with bladder exstrophy and high located umbilicus. It was a synonymous substitution in exon 10 (NM_006767.3, c.1146G>A, p.Ser382=, rs751444145, ExAC:ALL:A=0.0012%) which was predicted disease causing by in silico prediction softwares MutationTaster (Schwarz et al., 2010) and NNSPLICE (Reese, Eeckman, Kulp, & Haussler, 1997) since the distance to splice site is 4 bp. This prediction was not called by MaxEntScan (Yeo & Burge, 2004) or Human Splicing Finder (Desmet et al., 2009). This patient was adopted, and family segregation analysis was not possible.

### 3.4 Intracellular distribution of LZTR1

**Figure 1:**
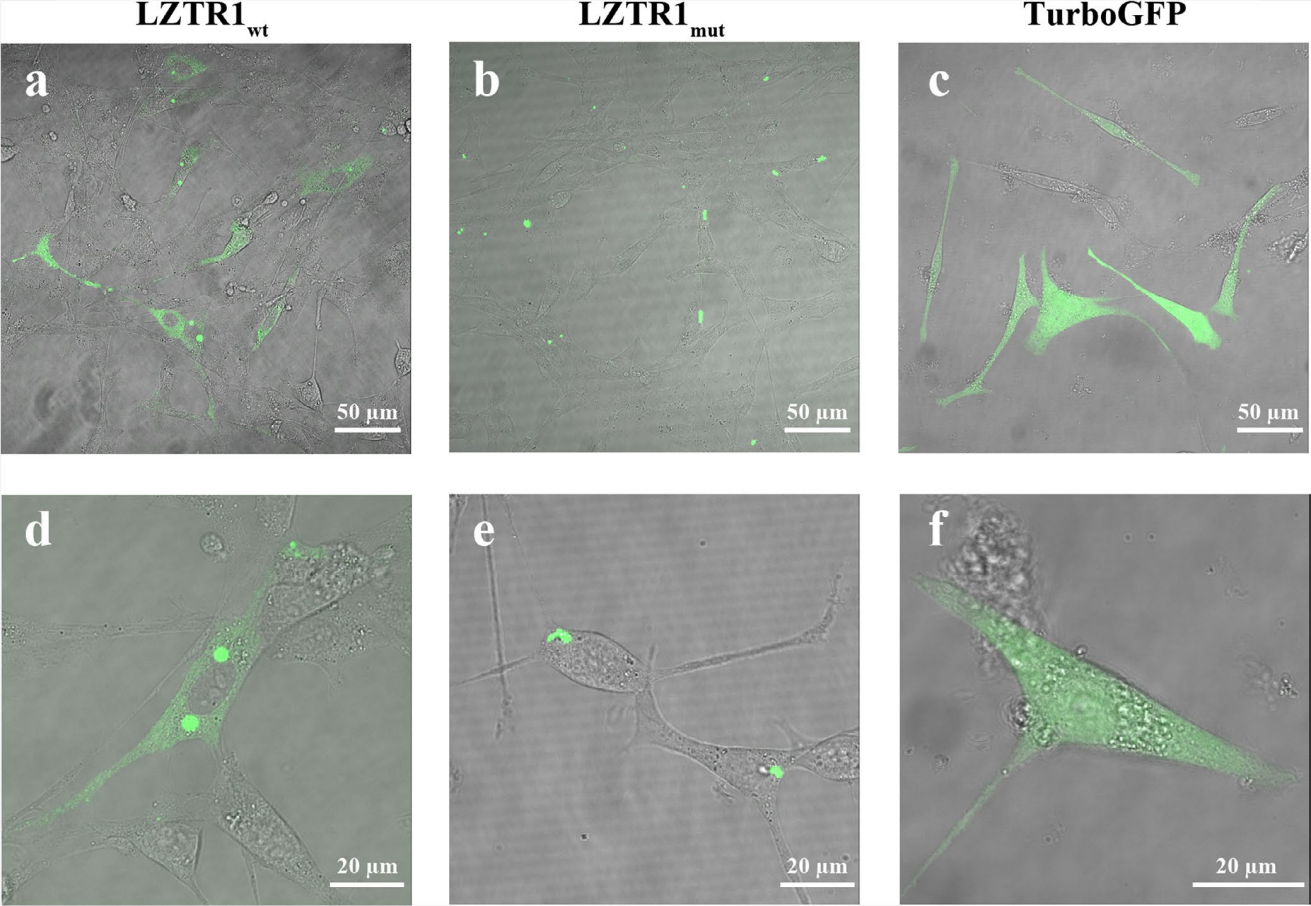
Lztr1_wt_ and Lztr1_mut_ differ in their intracellular distribution in live NIH 3T3 cells. CLSM images of the spatial distribution of Lztr1_wt_ (a and d), and Lztr1_mut_ (b and e), genetically fused with the reporter molecule TurboGFP, in live NIH 3T3 cells CLSM reveals that both Lztr1_wt_ and Lztr1_mut_ are localized in distinct, spatially confined structures that are associated with the endomembrane system (consistent with the Golgi), but Lztr1_wt_ was also observed in the cytoplasm, whereas Lztr1_mut_ was not. Images (c and f) show the uniform intracellular distribution of the fluorescence reporter, TurboGFP, alone

**Figure 2:**
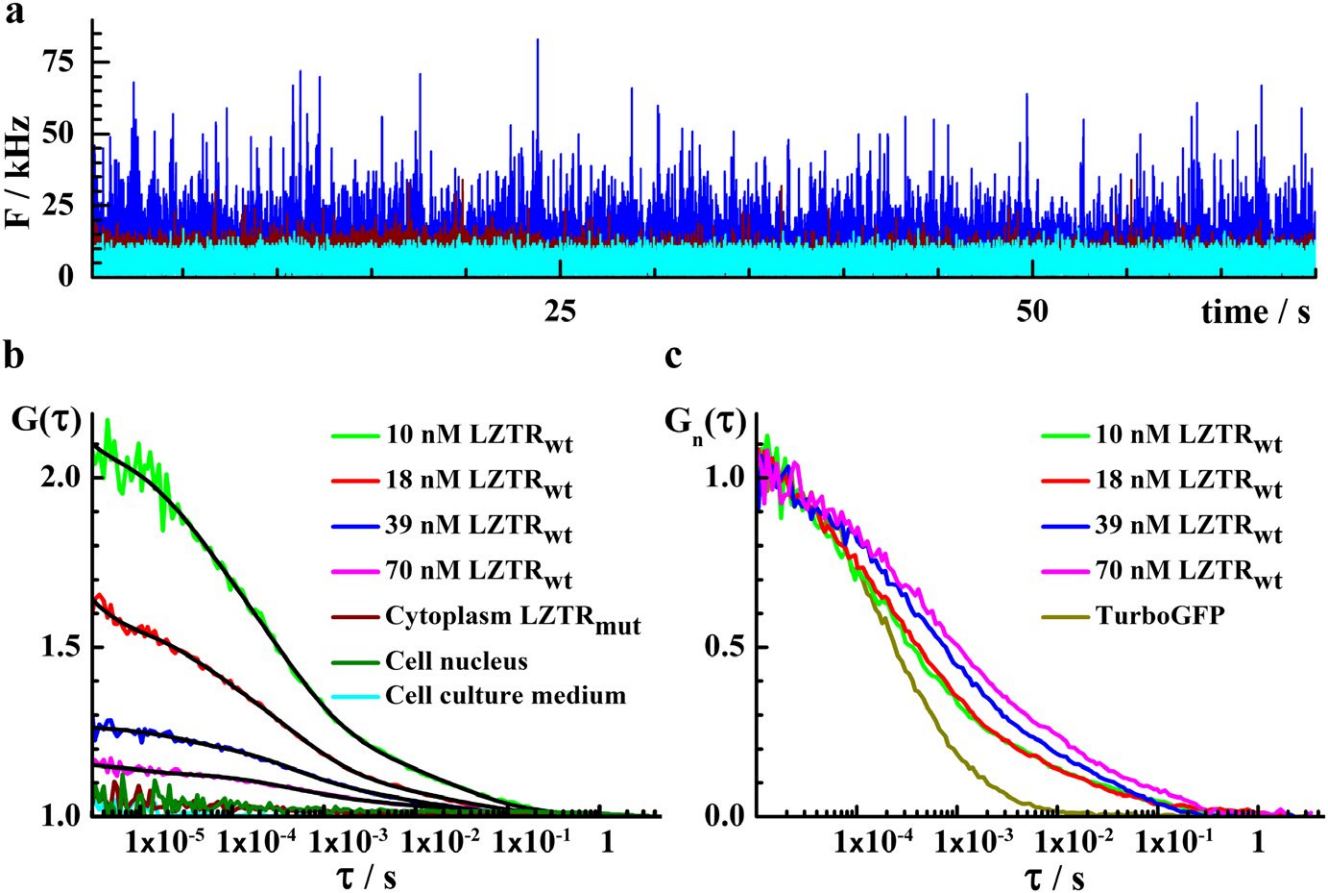
Lztr1_wt_ and Lztr1_mut_ differ in their intracellular distribution and Lztr1_wt_ mobility decay shifts to longer lag times with concentration, indicating complex formation in live NIH 3T3 cells. (a) Fluorescence intensity fluctuations recorded in the cytoplasm of live cells expressing Lztr1_wt_ (blue) or Lztr1_mut_ (wine). Fluorescence intensity fluctuations recorded in the cell culture medium (cyan) are shown for comparison. (b) Temporal autocorrelation curves (tACC) recorded in the cytoplasm of live cells expressing Lztr1_wt_ at different levels (10–70 nmol L^‐1^). Temporal autocorrelation analysis of fluorescence intensity fluctuations recorded in the cytoplasm of cells expressing Lztr1_mut_ showed that Lztr1_mut_ is not distributed in the cytoplasm (wine), outside of the very bright speckles observed by imaging. Nuclear localization was not observed; neither for Lztr1_wt_ nor for Lztr1_mut_ (dark green). (c) tACCs normalized to the same amplitude, G_n_(τ) = 1 at = τ10 µs, show that Lztr1_wt_ mobility is much slower compared to the mobility of TurboGFP (dark yellow), as evident from the shift of the characteristic decay time of the tACCs for Lztr1_wt_ towards longer lag times. FCS analysis also revealed that Lztr1_wt_ self‐assembles into larger supra‐molecular complexes when expressed at higher levels, as evident from the shift of the characteristic decay time of the tACC toward longer lag times. In addition, the contribution of the slower component increases for increasing Lztr1_wt_ concentration

Confocal laser scanning microscopy (CLSM) using avalanche photodiodes (APD) to allow single‐molecule sensitivity in the imaging mode (Vukojevic et al., 2008) and fluorescence correlation spectroscopy (FCS) (Elson, 2013; Vukojevic et al., 2005), a quantitative analytical technique with the ultimate single‐molecule sensitivity for measuring concentration and molecular diffusion, show in live cells that both Lztr1_wt_ and Lztr1_mut_ are localized in distinct, spatially confined structures that are associated with the endomembrane system (consistent with the Golgi), but Lztr1_wt_ was also observed in the cytoplasm, whereas Lztr1_mut_ was not (Figures 1,2).

Temporal autocorrelation analysis of time‐resolved fluorescence intensity fluctuations recorded in the cytoplasm of cells expressing Lztr1_wt_ or Lztr1_mut_ (Figure 2a) yielded temporal autocorrelation curves (tACC) for Lztr1_wt_, but not for Lztr1_mut_ (Figure 2b). Thus, FCS confirmed the results obtained by CLSM imaging (Figure 1), showing that Lztr1_mut_ is confined to the spatially constricted bright speckles observed by imaging, whereas this is not the case for Lztr1_wt_. Concentration of Lztr1_wt_ in the cytoplasm was measured to be 10–70 nmol L^‐1^ (Figure 2b). While Lztr1_wt_ and Lztr1_mut_ differ in their localization in the cytoplasm, nuclear localization was not observed, neither for Lztr1_wt_ nor for Lztr1_mut_. Furthermore, FCS showed that Lztr1_wt_ mobility in the cytoplasm is significantly slower than the mobility of TurboGFP, as evident from the shift of the characteristic decay time of the tACCs for Lztr1_wt_ toward longer lag times (Figure 2c). FCS also revealed that Lztr1_wt_ assembles into larger supra‐molecular complexes as its concentration in the cytoplasm increases, as evident from the increasing characteristic decay time of the tACCs which increases as the concentration of Lztr1_wt_ increases (Figure 2c).

## 4 DISCUSSION

The 22q11.2 microduplication was initially identified in a series of patients ascertained from overlapping features with the 22q11.2 deletion syndrome (Ensenauer et al., 2003) and is seen more often in this group of patients compared to healthy individuals (Cooper et al., 2011). Moreover, the 22q11.2 microduplication is the only recurrent and most frequent single genetic variant found in BEEC cases (Draaken et al., 2014,2010; Lundin et al., 2010; Pierquin & Uwineza, 2012) and compared to patients with intellectual disability and congenital defects it is eight times more common (2.61% vs. 0.32%). Array‐CGH analysis is today the first‐tier diagnostic tool for this later group of patients and, according to results presented here, should also be the first‐tier tool for genetic diagnosis of children born with BEEC.

Mutation screening of all protein coding genes in the 22q11.2 region in a microduplication negative BEEC cohort identified a variant of unclear significance in the *LZTR1 *gene (p.Ser698Phe). Functional evaluation of the variant in live cells using CLSM fluorescence imaging with single‐molecule sensitivity and FCS, showed that the intracellular localization, concentration and cytoplasmic mobility differ between the Lztr1_wt_ and Lztr1_mut_ in NIH 3T3 cells. CLSM showed that both Lztr1_wt_ and Lztr1_mut_ are localized in distinct, spatially confined structures that are associated with the endomembrane system (consistent with the Golgi), but Lztr1_wt_ was also observed in the cytoplasm whereas Lztr1_mut_ was not.

The *LZTR1* gene has been found to be involved in the development of schwannomatosis. Piotrowski et al. identified a germline heterozygous mutation in the *LZTR1* gene in 16 of 20 unrelated probands with schwannomatosis‐2 (OMIM 615670) implicating *LZTR1* as a tumor suppressor gene (Piotrowski et al., 2014). In affected members of five families with Noonan syndrome‐10 (OMIM 600574), Yamamoto et al. identified five different heterozygous missense mutations in the *LZTR1* gene following an autosomal dominant inheritance transmission in the families (Yamamoto et al., 2015). Recently, Steklov et al used a Lztr1 deletion mouse model and found that Lztr1 haploinsufficiency in mice recapitulates some of the Noonan syndrome phenotype that is, facial dysmorphism and heart malformation, whereas loss in Schwann cells drives dedifferentiation and proliferation (Steklov et al., 2018). For our two patients there is no record of schwannomatosis or clinical suspicion of Noonan syndrome. Mutations in the *LZTR1* gene have not previously been reported in BEEC cases. Draaken et al performed whole‐mount in situ hybridization of mouse embryos and investigated mice expression data for the 12 genes in the BEEC 22q11 phenocritical region with emphasis on the region of the ventrolateral trunk and the genital tubercle. The *LZTR1* was one of four genes that showed ubiquitous expression and was suggested potential candidate genes for the BEEC phenotype (Draaken et al., 2014).

In summary, mutations in the *LZTR1* gene do not seem to be a common cause of BEEC and even though we could not show any of the two variants to be a de novo event our functional evaluation suggests at least one variant to be damaging due to its loss of cytoplasmic expression. This warrants further studies into the role of the *LZTR1* gene in BEEC development are necessary. In conclusion, our study adds more evidence that the 22q11.2 microduplication is involved in the etiology of BEEC and that the *LZTR1* gene could be a prospective candidate gene.

## CONFLICT OF INTEREST

None declared.
